# Supplementary material for: Natural hybridization in heliconiine butterflies: the species boundary as a continuum
Source: BMC Evol Biol. 2007 Feb 23;7:28. doi: 10.1186/1471-2148-7-28 (PMC1821009; doi:10.1186/1471-2148-7-28)
Supplement: Additional File 1 — Hybrids between species of Heliconius and Eueides butterflies: a database. HTML file linking to database of all known wild-caught interspecific hybrid specimens in the Heliconiina, consisting of introductory text, a list of specimens, together with collection data and photographs of the specimens, and links to information about some artificial hybrids and mutants in the group. This is an edited copy of our online database of Heliconius hybrids [102]. To view database, download zip file and extract to a separate folder, then open index.html within that folder. [file 1471-2148-7-28-S1.zip › mavarez/c005.html]

butterfly c005


---


*Heliconius cydno cordula*
Venezuela: Táchira, San Cristobal, Jardín Botánico de Táchira, 1060m
Collected by Jesús Mavárez, October 2003
© James Mallet

Return to table of specimens

To next specimen
  
To previous specimen

**Last updated:** 2 November 2003

---
